# Gallic Acid Content in Taiwanese Teas at Different Degrees of Fermentation and Its Antioxidant Activity by Inhibiting PKCδ Activation: In Vitro and in Silico Studies

**DOI:** 10.3390/molecules21101346

**Published:** 2016-10-12

**Authors:** Teeradate Kongpichitchoke, Ming-Tzu Chiu, Tzou-Chi Huang, Jue-Liang Hsu

**Affiliations:** 1 Department of Agro-Industry, Assumption University, Bangkok 10240, Thailand; teeradate_abac@hotmail.com; 2 Taiwan Tea Experiment Station, Council of Agriculture, Taoyuan 326, Taiwan; mingtzu@ttes.gov.tw; 3 Department of Biological Science and Technology, National Pingtung University of Science and Technology, Pingtung 91201, Taiwan; tchuang@mail.npust.edu.tw

**Keywords:** Taiwanese tea, degree of fermentation, catechins, gallic acid, antioxidative activity, RAW264.7, protein kinase C, molecular docking

## Abstract

Teas can be classified according to their degree of fermentation, which has been reported to affect both the bioactive components in the teas and their antioxidative activity. In this study, four kinds of commercial Taiwanese tea at different degrees of fermentation, which include green (non-fermented), oolong (semi-fermented), black (fully fermented), and Pu-erh (post-fermented) tea, were profiled for catechin levels by using high performance liquid chromatography (HPLC). The result indicated that the gallic acid content in tea was directly proportional to the degree of fermentation in which the lowest and highest gallic acid content were 1.67 and 21.98 mg/g from green and Pu-erh tea, respectively. The antioxidative mechanism of the gallic acid was further determined by in vitro and in silico analyses. In vitro assays included the use of phorbol ester-induced macrophage RAW264.7 cell model for determining the inhibition of reactive oxygen species (ROS) production, and PKCδ and nicotinamide adenine dinucleotide phosphate (NADPH) oxidase subunit (p47) activations. The results showed that only at a concentration of 5.00 μM could gallic acid significantly (*p* < 0.05) reduce ROS levels in phorbol ester-activated macrophages. Moreover, protein immunoblotting expressed similar results in which activations of PKCδ and p47 were only significantly (*p* < 0.05) attenuated by 5.00 μM treatment. Lastly, in silico experiments further revealed that gallic acid could block PKCδ activation by occupying the phorbol ester binding sites of the protein.

## 1. Introduction

One of the most oft-consumed drinks in the world, especially in Asian countries [1], is tea [2,3]. Various beneficial health effects of teas, performed by bioactive components, were reported, such as lowering cholesterol [4] and low-density lipoprotein (LDL) content [5], reducing risk of type 2 diabetes [6,7], the risk of coronary artery disease [8], and strengthening immunity [4]. Teas can be classified according to their degree of fermentation. Non-fermented teas are usually immediately heated after the tea leaf harvesting process to inhibit enzymatic activities from both inside the leaves and microflora. In the case of fermented teas, the leaves are crushed and allowed to oxidize during the fermentation process. Although bioactive compounds in teas, such as catechin and its derivatives, have been reported for decades, compounds such as epicatechin (EC), epigallocatechin (EGC), epicatechin gallate (ECG), and epigallocatechin gallate (EGCG) undergo oxidative polymerization during fermentation, resulting in theaflavins [9]. The effect of the fermentation process on tea bioactive components changes and their antioxidative activity, as a consequence, have not been well documented.

Quantification of bioactive compounds in Pu-erh, green, oolong, and black teas were reported in which green tea had the highest amount of both total phenolics and catechins, whereas black tea and Pu-erh tea had the lowest levels of total phenolics and catechins, respectively [10]. With respect to the antioxidative activity of teas, catechin (C) and its derivatives; (+)-C, (−)-EC, (−)-EGC, (−)-ECG, (−)-EGCG, and (−)-gallocatechin gallate (GCG) play crucial roles for antioxidative activity [9]. Furthermore, polysaccharides in teas were also responsible for antioxidative activity in green, white, oolong, black, and dark green teas [11]. The total antioxidative activities of teas were reported in which green tea had the highest activity level, followed by oolong, black, and Pu-erh tea, in sequence [10]. Moreover, a comparison of antioxidants in tea was previously documented. The antioxidative activity, from high to low, was in the order of EGCG, EGC, procyanidin dimer, 3-galloyl-quinic acid, ECG, 1,2,6-tri-galloyl-glucose, and gallic acid [12].

Among other bioactive compounds in teas, the levels of gallic acid, which is a well-known catechin for its antioxidative activity, were found to vary in Chinese and Japanese commercial green, oolong, paochong, and Pu-erh teas. The highest gallic acid content was found in commercial Pu-erh tea (2.01 mg/100 mg) and the lowest amount in Chinese green tea (0.04 mg/100 mg) [13]. However, the changes in gallic acid in Taiwanese tea at different degrees of fermentation, its antioxidative activity and mechanisms in oxidative stress scenarios, still remains unclear.

Molecular docking, a kind of in silico assay, has been widely used to determine interactions between bioactive compounds with proteins to study their bioactivity. Gallic acid has been tested by docking assays with many proteins for various purposes, such as with lectin, to form a complex that can be used for drug delivery systems [14], and with HIV-1 protease to search for a possible phytochemical drug for HIV patients [15]. However, to the best of our knowledge, the interaction study between gallic acid with protein kinase C to study its antioxidative activity has never been reported. Furthermore, our previous study has proposed a RAW264.7 cell model, associated with an in silico assay, in which it had been proven for flavonoid antioxidative activity evaluation [16]. Therefore, we introduced gallic acid to this model to determine its antioxidative activity and mechanisms of action in which the results of this study would further suggest to us the validity of the model when it was applied to other compounds, rather than just flavonoids. In sum, we reported the effect of the fermentation process on bioactive compound levels in tea in this study. Further an investigation of the antioxidative activity was conducted on gallic acid, the bioactive compound mainly affected by the fermentation process, by using our previously proposed model.

## 2. Results and Discussion

### 2.1. Effect of the Fermentation Process on Catechins in Teas

Teas of four different degrees of fermentation, including green tea (non-fermented), oolong tea (semi-fermented), black tea (fully fermented), and Pu-erh tea (post-fermented) were investigated for their bioactive compound contents (Table 1). It was clear that the levels of gallic acid increased with the degree of fermentation, whereby the highest and lowest content were found in Pu-erh tea (21.98 mg/g) and green tea (1.67 mg/g), respectively. It was also found that catechin derivatives containing galloyl groups, i.e., ECG, EGCG, and GCG, in fermented teas (Pu-erh and black tea) were relatively lower than non-fermented (green tea) and semi-fermented teas (oolong tea). ECG in Pu-erh and black tea were less than one milligram, whereas almost two milligrams of the compound were found in green and oolong tea. Moreover, EGCG did not exist in both Pu-erh and black tea, while oolong and green tea contained EGCG at 13.34 and 14.03 mg/g, respectively. Furthermore, these two compounds without a galloyl group, i.e., EC, and EGC, in Pu-erh and black tea were also found at lower quantities than in green and oolong tea. EC and EGC content in Pu-erh and black teas were at least three and thirty times, respectively, less than oolong and green teas. Similar results for the gallic acid and catechin-derivative contents in teas were reported. Gallic acid in Pu-erh tea was five times higher than in green tea, while ECG, EGCG, GCG, EC, and EGC in Pu-erh tea were relatively lower than in green tea [17]. In addition, Lin et al. [13] also reported the composition of the bioactive compounds in 15 commercial Chinese green teas, 13 commercial Japanese green teas, and seven commercial Pu-erh tea products. The mean values of gallic acid content in the Chinese green tea, Japanese green tea, and Pu-erh tea products were 0.52, 0.23 and 1.49 mg/100 mg, respectively [13]. Moreover, ECG, EGCG, GCG, EC, and EGC quantities in the Pu-erh tea products were less than in the green tea products [13], which were in agreement with the results in this study. Therefore, the increase of gallic acid was likely because during the formation of theaflavins or other polyphenolic molecules by catechin derivatives in the tea fermentation process [9,18], where the gallic acid groups in catechin derivatives were probably released out, resulted in the reduction of EGCG and EGC.

### 2.2. DPPH Scavenging Activity of Teas and Inhibition of ROS Production by Gallic Acid in PMA-Activated RAW264.7 Cells

**Figure 1:**
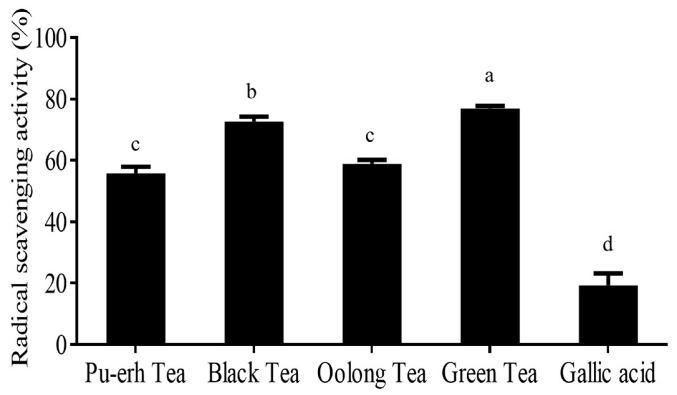
Radical scavenging activity of Taiwanese Pu-erh tea, black tea, oolong tea, and green tea. The test was performed by DPPH assay with three replicates. Different letters indicate the significant difference between groups (*p* < 0.05, Duncan test).

Regarding scavenging activity, the highest activity was found in green tea extract (76.83%), followed by black tea (72.61%), oolong tea (58.90%), and Pu-erh tea (55.77%), in sequence (Figure 1). This may be due to the fact that EGCG was found to be dramatically reduced along with the fermentation degree. EGCG contains high numbers of hydroxyl groups, which play an important role for scavenging free radicals. Thus, the fermentation process reduced the scavenging activity of tea. Additionally, the scavenging activity of gallic acid was also determined. It was found that gallic acid expressed scavenging activity at 19.33%.

**Figure 2:**
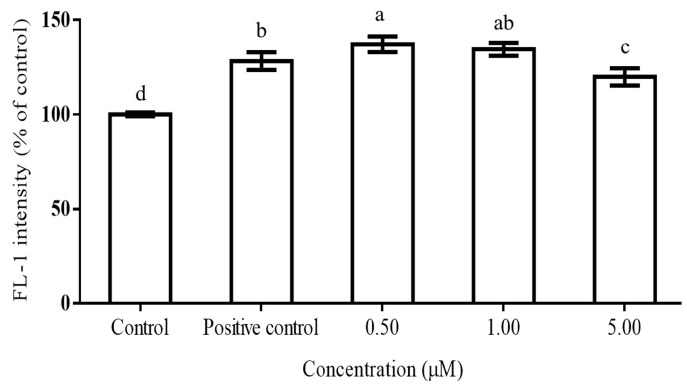
Inhibition of ROS production by gallic acid in phorbol ester-induced macrophage RAW264.7 cells. The cells were activated by PMA at 0.10 μg/mL concentration for 30 min (positive control). Gallic acid at different concentrations (0.50, 1.00 and 5.00 µM) were added to 1 × 10^6^ macrophage cells for 24 h prior to PMA activation. ROS levels were expressed as DCF intensity, measured in the FL-1 mode of flow cytometer, mean ± standard deviation. This assay was performed with three replicates for each treatment. Different letters indicate the significant difference in each group with other treatments (*p* < 0.05, Duncan test).

Gallic acid at five concentrations (0.5, 1.0, 5.0, 10, and 50 µM) were, firstly, tested for RAW264.7 cell cytotoxicity before ROS inhibition studies. Results showed that gallic acid at concentrations of 0.50, 1.00, and 5.00 μM had RAW264.7 cell survival percentages greater than 80% (data not shown). Thus, these three concentrations would be further used for ROS inhibition and protein immunoblotting assays. Figure 2 depicts inhibition activity of ROS production in the phorbol-12-myristate-13-acetate (PMA)-activated macrophage cells by gallic acid. We firstly found that PMA could significantly increase (*p* < 0.05) the FL-1 intensity to 128.29% of the control group, indicating that a remarkable amount of ROS had been produced by the cells. For gallic acid treatment with PMA-treated macrophages, gallic acid at a concentration of 0.50 µM significantly increased (*p* < 0.05) ROS production by 8.91% over the positive control group. An insignificant change in ROS from the 0.50 μM treatment was observed from the treatment of gallic acid at a concentration of 1.00 µM. However, a notable reduction of ROS, compared with the positive control, was only found from a concentration of 5.00 µM, which could reduce 8.38% of the ROS level from the positive control group. This was because gallic acid could induce ROS production and consequently lead to cell apoptosis or anti-proliferation in many cancer cell lines [19,20,21]. However, reductions of the superoxide from induced cells, which were similar to our study, were also reported. Gallic acid could reduce ROS levels in SH-SY5Y cells induced by 6-hydrodopamine autoxidation [22]. In addition, gallic acid was tested for ROS production in neutrophils, a main producer of superoxide, in which IC_50_ value of 0.40 µg/mL was reported for ROS inhibition in PMA-treated neutrophils cells [23], and ROS significantly reduced by gallic acid were found at concentrations of 50–100 μM in LPS-induced cells [24]. Therefore, our study suggested that gallic acid at low concentrations (0 and 0.50 µM) similarly behaved as ROS stimulators, which occurred with cancer cells, but at high concentration (1.00 µM) gallic acid changed its function to that of an antioxidant. The IC_50_ value of gallic acid in this study was 19.80 µM.

### 2.3. Inhibition of Phosphorylation of PKCδ and p47^phox^ Proteins

**Figure 3:**
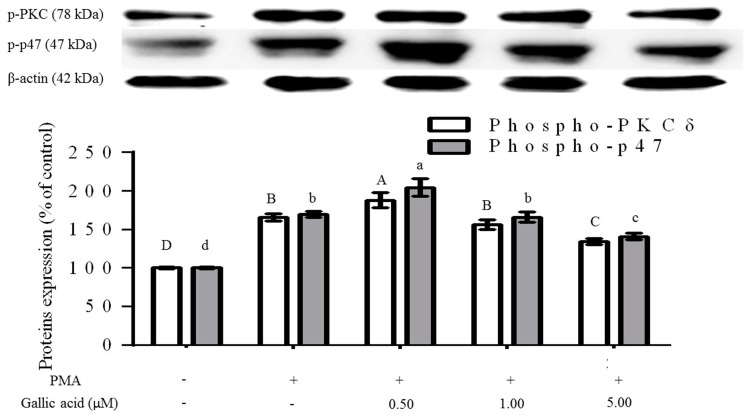
Gallic acid inhibited PKCδ and p-47^phox^ activation. Gallic acid at different concentrations (0.50, 1.00 and 5.00 µM) were treated to 1 × 10^6^ cells RAW264.7 macrophage cells for 24 h prior to 30 min of PMA activation. This assay was performed with three replicates for each treatment. The values were expressed as mean ± standard deviation. Different letters indicate the significant difference in each group with other treatments (*p* < 0.05, Duncan test).

From the previous section we found that gallic acid at high enough concentration could reduce ROS production in PMA-activated RAW264.7 cells. Further investigation on the inhibition mechanism between the compounds with PKCδ and NADPH oxidase subunit, p47^phox^, were conducted by protein immunoblotting. It was found that PMA induced phosphorylation of PKC and p47^phox^ (Figure 3). Moreover, both signalling proteins were significantly activated and translocated (*p* < 0.05) to a lipid raft layer in which intensities of both proteins at the cell membrane were 65.71% and 69.46% higher than the control group. This caused an increase of ROS production in the positive control group in the ROS inhibition assay. Furthermore, the results confirmed that gallic acid attenuated phosphorylation of both PKCδ and p47^phox^ (Figure 3). An increase of gallic acid content in PMA-activated RAW264.7 would result in a decrease of phosphor-PKCδ and p47^phox^ intensities in which phosphor-PKCδ intensities from 0.50, 1.00 and 5.00 µM gallic acid were 113.25%, 94.15% and 80.92%, respectively, of the positive control group, whereas phosphor-p47^phox^ intensities were 120.67%, 97.83% and 83.00%, correspondingly. Results from western blotting experiments were in accordance with superoxide inhibition assays. At low concentrations of gallic acid (0.50 µM), significant activation of key proteins mediating ROS production over the positive control was found. An important reduction of the activation was accomplished when the gallic acid concentration was raised up to 5.00 µM, while an insignificant change was performed by the middle concentration in this study (1.00 µM). According to our previously proposed cell model [16], inhibition of the phosphorylation of both PKCδ and p47^phox^ would result in the reduction of oxidative stress induced by PMA. Nonetheless, other bioactive compounds in tea, not only gallic acid, could reduce oxidative stress, as polyphenols containing galloyl groups have also been documented for their ability to suppress NADPH oxidase activation induced by PMA [25]. To the best of our knowledge, the inhibition of the phosphorylation of PKCδ and p47^phox^ in PMA-activated cells by gallic acid has never been reported.

### 2.4. Molecular Docking

**Figure 4:**
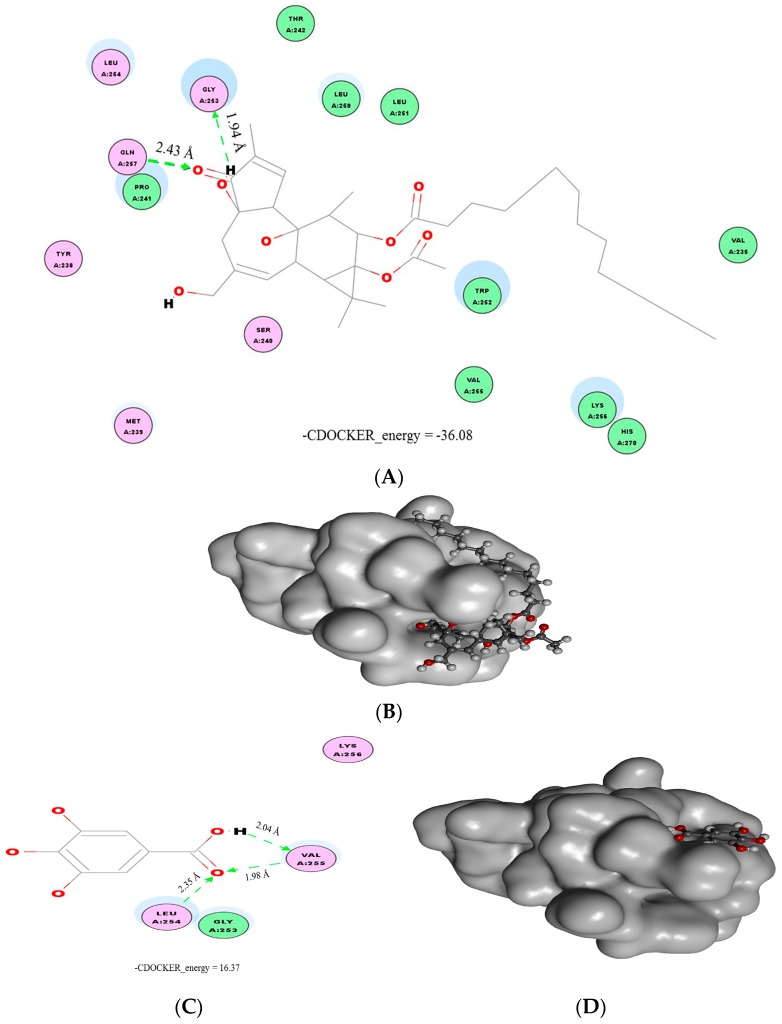
Interactions between phorbol ester and gallic acid with the C1B domain phorbol ester binding site of PKCδ. Molecular docking characteristics between ligands: phorbol ester (**A** and **B**) and gallic acid (**C** and **D**) were determined by the Accelrys Discovery Studio Visualizer software. Hydrogen bonding between phorbol ester (**A**) and gallic acid (**C**) are colored in green, and numbers above each bond represent its length. The best alignment of phorbol ester (**B**) and gallic acid (**D**), according to −CDOCKER_energy values, binding with the three-dimensional surface of the PKCδ C1B domain at the phorbol ester binding site (painted with transparent grey) is shown. Molecular structures are presented in ball and stick style. Oxygen, carbon, and hydrogen atoms are painted in red, dark grey, and light grey, respectively.

Results from the protein immunoblotting suggested that gallic acid could inhibit phosphorylation of PKCδ and p47^phox^ similarly to flavonoids [16]. Thus, phorbol ester (phorbol-13-*O*-acetate) was, first, docked with the phorbol ester binding site of PKCδ to determine the activation mechanisms (Figure 4A,B). Although the binding characteristics between the phorbol ester and the PKCδ C1B domain had been reported, our study results showed differences. It was indicated that Thr-242, Leu-251, and Gly-253 amino acids of the C1B domain formed hydrogen bonds with the phorbol ester at the C-4 and C-20 hydroxyl groups and the C-3 carbonyl group [26,27]. Our study revealed that hydrogen bridges were formed between Gly-253 and the C-4 hydroxyl, and Gln-257 and the C-3 carbonyl functional group. It seems that Gly-253 plays a crucial role for phorbol ester [26] and flavonoids [16] to interact with PKCδ at the phorbol ester binding sites. In contrast, other kinds of binder could bind with other amino acid residues, such as Leu-251 and Gln-257 for curcumin [27], and Leu-254 and Val-255 for gallic acid in this study. Then, gallic acid was studied for interactions with the proteins (Figure 4C,D). We found that three possible hydrogen bonds were formed between gallic acid at the carboxylic acid part and the C1B domain at Leu-254 and Val-255. Considering −CDOCKER_energy, phorbol ester and gallic acid had values of −36.08 and 16.37, respectively. These values reflected that gallic acid had better binding affinity with PKCδ over phorbol ester. This might be because gallic acid uses its carboxylic group rather than three hydroxyl groups on the carbon backbone ring to attach with the binding site, creating stronger interactions than the phorbol ester, which used carbonyl and hydroxyl groups.

Interestingly, even though gallic acid had several hydroxyl groups available for bonding interactions with PKCδ, as occurred with flavonoids [16], none of them succeeded in forming interactions. It has been discussed that the *ortho*-arrangement of hydroxyl groups could form two hydrogen bridges with the site, allowing stronger interaction than *meta*-hydroxyl groups, which have only single hydrogen bonding [16]. In this research, gallic acid, containing three additional hydroxyl groups, in both *ortho*- and *meta*-arrangements, could not use hydroxyl groups to interact with the binding site. This emphasized the importance of additional hydroxyl groups’ positions to the benzene ring for binding with the phorbol ester binding site of PKCδ. Evidence shows that gallic acid has a smaller size than the flavonoids which, consequently, allows great flexibility for binding with the site. Therefore, for two substituted hydroxyl groups to the benzene ring *ortho*-arrangement was better in terms of interaction with the binding site, compared with the *meta*-arrangement. On the other hand, three consecutive additional hydroxyl groups were not suitable to function as interaction sites with the PKCδ C1B domain.

## 3. Materials and Methods

### 3.1. Chemical and Raw Materials

Methanol was purchased from J.T. Baker (Center Valley, PA, USA). Formic acid, catechol, PMA, 2′,7′-dichlorodihydrofluorescein diacetate (DCFH-DA), dimethyl sulphoxide (DMSO), 2,2-diphenyl-1-picrylhydrazyl (DPPH), butylated hydroxyanisole (BHA), gallic acid, EGC, C, EGCG, EC, GCG, and ECG were brought from Sigma-Aldrich (St. Louis, MO, USA). Antibiotics and fetal bovine serum (FBS) were obtained from Biological Industries (Haemek, Israel). Dulbecco’s modified Eagle’s medium (DMEM) and 3-(4,5-dimethylthiazol-2-yl)-2,5-diphenyltetrazolium bromide (MTT) were bought from Gibco BRL (Grand Island, NY, USA) and AppliChem (Saxony-Anhalt, Germany), respectively. Commercial green, black, oolong, and Pu-erh tea powders were obtained from the Tea Research and Extension Station, Council of Agriculture, Taiwan.

### 3.2. Tea Extraction

Two grams of each tea sample were extracted with double-distilled water (50 mL) at 70 °C for 30 min. The tea extracts were then filtered through a 0.22 µm filter membrane. The filtrate was stored at 4 °C for further experiments.

### 3.3. HPLC Analysis

High performance liquid chromatography (HPLC) analysis was performed by using L-7100, L-7200 and L-7420, Hitachi HPLC system (Hitachi, Tokyo, Japan) equipped with a reverse phase-C18 column (250 × 4.6 mm, 5 µm, MightysilKanto Chemical, Tokyo, Japan). The sample load was 10 µL. Methanol at concentrations of 20% and 100%, both in 0.3% formic acid, were used as solvents A and B, respectively. The gradient program was as follows: 0% B at the initial stage and maintained for 10 min; 0%–10% B for 10–25 min; 10%–30% B for 25–60 min; 30% B for 60–75 min; 30%–0% B for 75–76 min; and 0% B for 76–85 min. Measurement of bioactive compounds was done by using a UV-VIS detector at 280 nm. Quantification of the compounds was calculated by using standard catechins, gallic acid, and catechol as internal standards [28].

### 3.4. DPPH Radical Scavenging Assay

Radical scavenging of tea extracts were determined with the method reported by Kongpichitchoke et al. [16]. In short, 250 µL of 1 mM DPPH solution was reacted with 2 mL of tea extracts or gallic acid at a concentration of 10 µM. Then, the mixture was mixed well and placed in the dark for 30 min. Finally, the absorbance value was measured at 517 nm, and scavenging activity was calculated in which BHA was used as standard. The DPPH scavenging activity of each tea extract was determined in triplicate.

### 3.5. Cell Culture

Murine macrophage cells (RAW264.7) were provided by the Bioresource Collection and Research Center, Hsinchu, Taiwan. DMEM medium, added with 0.5% antibiotics (*v*/*v*) and 10% fetal bovine serum, was used to culture the cells in a sterilized Petri dish (Corning, NY, USA). The cells were cultivated in an incubator at 37 °C under 5% CO_2_ and 95% air with humidification, and subcultured every two days. Before cell treatment with gallic acid, cells were counted in a counting chamber, and seeded at a concentration 1 × 10^6^ cells/well into 96 well plates for cell cytotoxicity assay or six well plates for ROS inhibition and protein immunoblotting assays. After seeding the cells, the cell container was placed in the incubator for 12 h for acclimation and followed with gallic acid treatments.

### 3.6. Cell Viability Test

A MTT assay was performed to determine cell cytotoxicity effects of gallic acid. RAW264.7 cells at a concentration of 1 × 10^6^ cells/well were cultured in 96 well plates as described in the cell culture and treatments section. Then, the cell culture was treated with gallic acid at final concentrations of 0.50, 1.00, 5.00, 10.0, and 50.0 μM in appropriate amounts of DMSO and placed in the incubator for 24 h. The medium was removed and MTT solution was added into each well. After that, the plate was placed in the dark at room temperature for three hours. DMSO was added and reacted to dissolve the purple formazan compound for 30 min. Finally, the optical density (OD) was observed at 570 nm by an ELISA reader (VersaMax Microplate Reader, Molecular Devices, Sunnyvale, CA, USA). Ten replications for each gallic acid concentration were measured for the OD and each treatment was repeated three times.

### 3.7. ROS Inhibition and IC_50_ Determination

Inhibition of ROS by gallic acid was analyzed by using a flow cytometer (FACSCaliburTM, BD Biosciences, Mississauga, ON, Canada). After acclimation of the cell culture for 12 h in six well plates, gallic acid, at its final concentrations of 0.50, 1.00, and 5.00 µM, in DMEM medium, was added into each well for 24 h with the treatment of DCFH-DA (final concentration of 10.0 µM) in the last 30 min. Then, the medium was discarded. The cells were washed by phosphate-buffered saline (PBS) before cell collection by using a cell scraper. ROS production was then induced by PMA (final concentration of 0.10 μg/mL) for 30 min. A cell pellet was obtained by centrifuging the cell suspension at 500× *g* (4 °C) for five minutes. After that, the cell pellet was re-suspended in PBS and the intensity of dichlorodihydrofluorescein (DCF) was determined by the FL-1 channel of the flow cytometer. Area gating was done via a dot plot under the E-1 mode to achieve data collection from 90% of the cell sample in which 10,000 gated cells were measured for DCF intensity. The IC_50_ was the gallic acid concentration required to inhibit half or 50% of the PMA-induced ROS production, which was calculated using linear regression of the ROS inhibition (%) from three different concentrations of gallic acid. Each treatment was repeated five times.

### 3.8. Protein Immunoblotting Assay

RAW264.7 cells were treated with gallic acid for 24 h at the same concentration as in the ROS inhibition assay. In the last 30 min of gallic acid treatment, PMA, at its final concentration (0.10 μg/mL), was added to the cell culture. After that, the cells were washed by PBS before cell harvesting and cell membrane proteins were extracted and quantified using Mem-PER Plus Membrane Protein extraction kit and bicinchoninic acid (BCA) protein assay kit standardized with bovine serum albumin (Thermo Scientific, Waltham, MA, USA), respectively. Membrane protein extracts were equally loaded at 30.0 µg into each lane of 8% sodium dodecyl sulfate (SDS)-polyacrylamide gel. Proteins were separated by applying 110 volts for 110 min. The transfer process of proteins from the gel to polyvinylidene-fluoride (PVDF) membrane (PerkinElmer, Waltham, MA, USA) was made at 250 mA for one hour in a refrigerator. Proteins on the membrane were subjected to blocking buffer for one hour and primary antibodies, β-actin (42 kDa) phosphor-PKCδ Thr505 (78 kDa) (Cell Signaling, Danvers, MA, USA) and phosphor-p47 (47 kDa, Assay Biotech, Sunnyvale, CA, USA), for 16–18 h at 4 °C. The primary antibodies were then washed out from the membrane by trist-buffered saline with tween 20 (TBST) buffer for one hour. After that, the membrane was subjected to secondary antibodies (Merck Millipore, Billerica, MA, USA) for one hour at room temperature, the membranes were cleaned again by TBST buffer for one hour, and the protein bands were revealed with an enhanced chemiluminescence reagent (ECL, Advansta, Menlo Park, CA, USA).

### 3.9. Molecular Docking

Molecular docking between the PKCδ phorbol ester binding site C1B domain and gallic acid was studied in Accelrys Discovery Studio Visualizer program (version 3.0, Biovia, San Diego, CA, USA). The C1B domain 3D structure (database code 1PTQ) was downloaded from the Research Collaboratory for Structural Bioinformatics Protein Data Bank (RCSB PDB). Phorbol ester and gallic acid molecules were downloaded from the ZINC database [29]. Docking preparation processes included the removal of all small molecules surrounding the PKCδ structure, molecules’ energy minimization by the CHARMm program, and the binding site definition of the C1B domain at the glycine 253 coordinate (x, 7.104; y, 24.326; and z, 27.682). The CDOCKER tool in the software was used to determine the 10 best positions for molecular docking based on the −CDOCKER_energy.

### 3.10. Statistical Analysis

All results were analyzed for analysis of variance (ANOVA) using the Statistical Package for the Social Sciences program (SPSS, version 19. SPSS Inc., Endicott, NY, USA). Significant differences between means were determined by Duncan’s multiple-range test or the Student’s *t*-test (*p* < 0.05).

## 4. Conclusions

**Figure 5:**
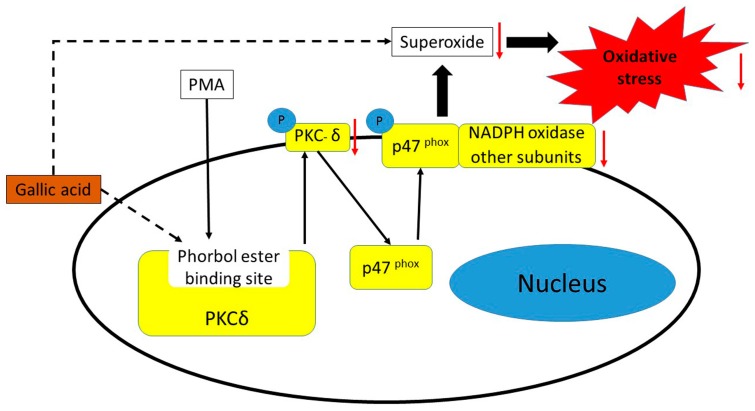
Antioxidative activity of gallic acid in phorbol ester-induced RAW264.7 macrophages (dashed lines). Gallic acid can reduce oxidative stress by directly neutralizing free radicals, and occupying the phorbol ester binding site of PKCδ.

The catechins in tea were greatly affected by the tea fermentation process, whereby gallic acid was found to be significantly released after enzymatic degradation during the fermentation process. Furthermore, gallic acid was evaluated for its antioxidative activity in a phorbol ester-induced oxidative stress cell model. Both in vitro and in silico analyses suggested that gallic acid could reduce the oxidative stress by, first, neutralizing free radicals and, second, occupying the binding site of PKCδ instead of phorbol ester, and consequently suppressing activation of PKCδ and the phosphorylation of p47^phox^ resulting in ROS reduction (Figure 5). Lastly, the cell model was proven to be able to determine the antioxidative activity for not only flavonoids, but also other bioactive compounds.

## Figures and Tables

**Table 1:** Analysis of major bioactive compounds in Taiwan teas. GA: gallic acid, EGC: epigallocatechins, C: catechin, EGCG: epigallocatechin gallate, EC: epicatechin, GCG: gallocatechin gallate, and ECG: epicatechin gallate.

Sample	Bioactive Compound Content (mg/g)						
	GA	EGC	C	EGCG	EC	GCG	ECG
Pu-erh Tea	21.98 ± 1.03	0.02 ± 0.01	0.50 ± 0.09	0	1.45 ± 0.33	0.49 ± 0.12	0.22 ± 0.02
Black Tea	6.09 ± 0.06	0.07 ± 0.01	0.55 ± 0.03	0	0.95 ± 0.35	1.00 ± 0.05	0.45 ± 0.10
Oolong Tea	1.92 ± 0.56	2.33 ± 0.50	1.09 ± 0.47	13.34 ± 3.87	4.54 ± 0.14	1.04 ± 0.34	2.42 ± 0.71
Green Tea	1.67 ± 0.06	3.46 ± 0.06	2.30 ± 0.08	14.03 ± 0.89	8.86 ± 0.09	1.21 ± 0.04	1.89 ± 0.07

## References

[1] Tseng H.C., Wang C.J., Cheng S.H., Sun Z.J., Chen P.S., Lee C.T., Lin S.H., Yang Y.K., Yang Y.C. (2014). Tea-drinking habit among new university students: Associated factors. Kaohsiung J. Med. Sci..

[2] Lu C.M., Lan S.J., Lee Y.H., Huang J.K., Huang C.H., Hsieh C.C. (1999). Tea consumption: Fluid intake and bladder cancer risk in southern Taiwan. Urology.

[3] Huang Y., Xiao X., Cong L., Wu M., Huang Y., Yao Y. (2016). A fermented tea with high levels of gallic acid processed by anaerobic solid-state fermentation. LWT-Food Sci. Technol..

[4] Yang C.S., Lambert J.D. (2011). Research on tea and health. Pharmacol. Res..

[5] Kim A., Chiu A., Barone M.K., Avino D., Wang F., Coleman C.I., Phung O.J. (2011). Green tea catechins decrease total and low-density lipoprotein cholesterol: A systematic review and meta-analysis. J. Am. Diet. Assoc..

[6] Huxley R., Lee C.M.Y., Barzi F., Timmermeister L., Czernichow S., Perkovic V., Grobbee D.E., Batty D., Woodward M. (2009). Coffee, decaffeinated coffee, and tea consumption in relation to incident type 2 diabetes mellitus: A systematic review with meta-analysis. Arch. Intern. Med..

[7] Jing Y., Han G., Hu Y., Bi Y., Li L., Zhu D. (2009). Tea consumption and risk of type 2 diabetes: A meta-analysis of cohort studies. J. Gen. Intern. Med..

[8] Wang Z.M., Zhou B., Wang Y.S., Gong Q.Y., Wang Q.M., Yan J.J., Gao W., Wang L.S. (2011). Black and green tea consumption and the risk of coronary artery disease: A meta-analysis. Am. J. Clin. Nutr..

[9] Kim Y., Goodner K.L., Park J.D., Choi J., Talcott S.T. (2011). Changes in antioxidant phytochemicals and volatile composition of *Camellia sinensis* by oxidation during tea fermentation. Food Chem..

[10] Duh P.D., Yen G.C., Yen W.J., Wang B.S., Chang L.W. (2004). Effects of Pu-erh tea on oxidative damage and nitric oxide scavenging. J. Agric. Food Chem..

[11] Zhao Z.Y., Huangfu L.T., Dong L.L., Liu S.L. (2014). Functional groups and antioxidant activities of polysaccharides from five categories of tea. Ind. Crop. Prod..

[12] Zhang Y., Li Q., Xing H., Lu X., Zhao L., Qu K., Bi K. (2013). Evaluation of antioxidant activity of ten compounds in different tea samples by means of an on-line HPLC-DPPH assay. Food Res. Int..

[13] Lin J.K., Lin C.L., Liang Y.C., Shiau S.Y.L., Juan I.M. (1998). Survey of catechins, gallic acid, and methylxanthines in green, Oolong, Pu-erh, and black Teas. J. Agric. Food Chem..

[14] Surya S., Geethanandan K., Sadasivan C. (2016). Gallic acid binding to *Spatholobus parviflorus* lectin provides insight to its quaternary structure forming. Int. J. Biol. Macromol..

[15] Singh A., Pal T.K. (2015). Docking analysis of gallic acid derivatives as HIV-1 protease inhibitors. Int. J. Bioinform. Res. Appl..

[16] Kongpichitchoke T., Hsu J.L., Huang T.C. (2015). Number of hydroxyl groups on the B‑Ring of flavonoids affects their antioxidant activity and interaction with phorbol ester binding site of PKCδ C1B Domain: In vitro and in silico studies. J. Agric. Food Chem..

[17] Zhang L., Li N., Ma Z.Z., Tu P.F. (2011). Comparison of the chemical constituents of aged Pu-erh tea, ripened Pu-erh Tea, and other teas using HPLC-DAD-ESI-MS^n^. J. Agric. Food Chem..

[18] Zuo Y., Chen H., Deng Y. (2002). Simultaneous determination of catechins, caffeine and gallic acids in green, Oolong, black and pu-erh teas using HPLC with a photodiode array detector. Talanta.

[19] You B.R., Park W.H. (2010). Gallic acid-induced lung cancer cell death is related to glutathione depletion as well as reactive oxygen species increase. Toxicol. In Vitro.

[20] Weng C.J., Yen G.C. (2012). Chemopreventive effects of dietary phytochemicals against cancer invasion and metastasis: Phenolic acids, monophenol, polyphenol, and their derivatives. Cancer Treat. Rev..

[21] Verma S., Singh A., Mishra A. (2013). Gallic acid: Molecular rival of cancer. Environ. Toxicol. Pharmacol..

[22] Lu Z., Nie G., Belton P.S., Tang H., Zhao B. (2006). Structure-activity relationship analysis of antioxidant ability and neuroprotective effect of gallic acid derivatives. Neurochem. Int..

[23] Boly R., Franck T., Kohnen S., Lompo M., Guissou I.P., Dubois J., Serteyn D., Mickalad A.M. (2015). Evaluation of antiradical and anti-inflammatory activities of ethyl acetate and butanolic subfractions of *Agelanthus dodoneifolius* (DC.) Polhill & Wiens (Loranthaceae) using equine myeloperoxidase and both PMA-activated neutrophils and HL-60 cells. Evid. Based Complement. Altern. Med..

[24] Haute G.V., Caberlon E., Squizani E., Mesquita F.C., Pedrazza L., Martha B.A., Melo D.A.S., Cassel E., Czepielewski R.S., Bitencourt S. (2015). Gallic acid reduces the effect of LPS on apoptosis and inhibits the formation of neutrophil extracellular traps. Toxicol. In Vitro.

[25] Lin J.K., Chen P.C., Ho C.T., Shian S.Y.L. (2002). Inhibition of xanthine oxidase and NADPH oxidase by tea polyphenols. Free Radicals in Food.

[26] Zhang G., Kazanietz M.G., Blumberg P.M., Hurley J.H. (1995). Crystal structure of the Cys2 activator-binding domain of protein kinase Cδ in complex with phorbol ester. Cell.

[27] Majhi A., Rahman G.M., Panchal S., Das J. (2010). Binding of curcumin and its long chain derivatives to the activator binding domain of novel protein kinase C. Bioorg. Med. Chem..

[28] Mizukami Y., Sawai Y., Yamaguchi Y. (2007). Simultaneous analysis of catechins, gallic Acid, strictinin, and purine alkaloids in green tea by using catechol as an internal standard. J. Agric. Food Chem..

[29] Irwin J.J., Sterling T., Mysinger M.M., Bolstad E.S., Coleman R.G. (2012). ZINC: A Free Tool to Discover Chemistry for Biology. J. Chem. Inf. Model..

